# Challenges and Adaptation of a European Influenza Vaccine Effectiveness Study Platform in Response to the COVID-19 Emergence: Experience from the DRIVE Project

**DOI:** 10.3390/ijerph18031058

**Published:** 2021-01-25

**Authors:** Antonio Carmona, Cintia Muñoz-Quiles, Anke Stuurman, Alexandre Descamps, Ainara Mira-Iglesias, Laurence Torcel-Pagnon, Javier Díez-Domingo

**Affiliations:** 1 Fundación para el Fomento de la Investigación Sanitaria y Biomédica de la Comunidad Valenciana (Fisabio), Avenida Cataluña 21, 46020 Valencia, Spain; munoz_cin@gva.es (C.M.-Q.); Ainara.Mira@fisabio.es (A.M.-I.); Javier.Diez@fisabio.es (J.D.-D.); 2 P-95 CVBA, Koning Leopold III laan 1, 3001 Heverlee, Belgium; Anke.stuurman@p-95.com; 3 INSERM CIC 1417, Assistance Publique-Hôpitaux de Paris, Université de Paris, Hôpital Cochin, 75005 Paris, France; alexandre.descamps@aphp.fr; 4 Sanofi-Pasteur SA (SP), 14 Espace Henry Vallée, 69007 Lyon, France; laurence.pagnon@sanofi.com

**Keywords:** COVID-19, influenza, vaccination, vaccine effectiveness, test-negative design

## Abstract

The Development of Robust and Innovative Vaccine Effectiveness (DRIVE) project is a public–private partnership aiming to build capacity in Europe for yearly estimation of brand-specific influenza vaccine effectiveness (IVE). DRIVE is a five-year project funded by IMI (Innovative Medicines Initiative). It was initiated as a response to the guidance on influenza vaccines by EMA (European Medicines Agency), which advised vaccine manufacturers to work with public health institutes to set up a joint IVE study platform. The COVID-19 pandemic reached Europe in February 2020 and overlapped with the 2019/2020 influenza season only in the last weeks. However, several elements of the DRIVE study network were impacted. The pandemic specifically affected the study sites’ routines and the subsequent assessment of the 2019/20 influenza season. Moreover, the current social distancing measures and lockdown policies across Europe are expected to also limit the circulation of influenza for the 2020/21 season, and therefore the impact of COVID-19 will be higher than in the season 2019/20. Consequently, DRIVE has planned to adapt its study platform to the COVID-19 challenge, encompassing several COVID-19 particularities in the study procedures, data collection and IVE analysis for the 2020/21 season. DRIVE will study the feasibility of implementing these COVID-19 components and establish the foundations of future COVID-19 vaccine effectiveness studies.

## 1. Introduction

### 1.1. DRIVE Project, a Public–Private Partnership to Monitor Influenza Vaccine Effectiveness

The ‘Development of Robust and Innovative Vaccine Effectiveness’ (DRIVE) (https://www.drive-eu.org/) project was launched as a five-year public–private partnership in order to respond to a guideline issued in 2017 by the European Medicines Agency (EMA), requesting vaccine manufacturers to provide a yearly brand-specific effectiveness evaluation for all influenza vaccines as part of their post-licensure regulatory requirements [1].

For this purpose, the European Commission, through the Innovative Medicines Initiative (IMI), supported the DRIVE project, a pioneer public–private partnership (PPP) that advances European cooperation in influenza vaccine effectiveness (IVE) studies. The data generated through DRIVE are expected to increase the understanding of influenza vaccine effectiveness, lead to enhanced monitoring of influenza vaccine performance by public health institutes and allow manufacturers to fulfil regulatory requirements. 

### 1.2. DRIVE Influenza Vaccine Effectiveness Studies through the Years

DRIVE’s main objective in its first pilot season (2017/2018) was to establish and test the feasibility of a new multi-country platform using a limited number of sites (five study sites from four different countries, see Table 1) [2]. Although the influenza season was severe, precise IVE estimates were not obtained due in part to the limited number of participating study sites and the resultant limited sample size. However, DRIVE succeeded in setting up the IVE study platform and implementing DRIVE generic protocols and standard operating procedures across the different sites. 

For the 2018/2019 season, the network expanded from 5 to 13 sites from seven different European countries (Table 1). DRIVE protocols were further harmonized, the statistical analysis plan was improved, and age- and setting-stratified IVE estimates were calculated [2,3]. Furthermore, a post hoc analysis of the 2018/2019 data allowed for the simplification of the confounders that were considered for the following season, 2019/2020, permitting the participation of study sites that had limited data on confounders and avoiding potential over-adjustment [4,5].

**Figure 1:**
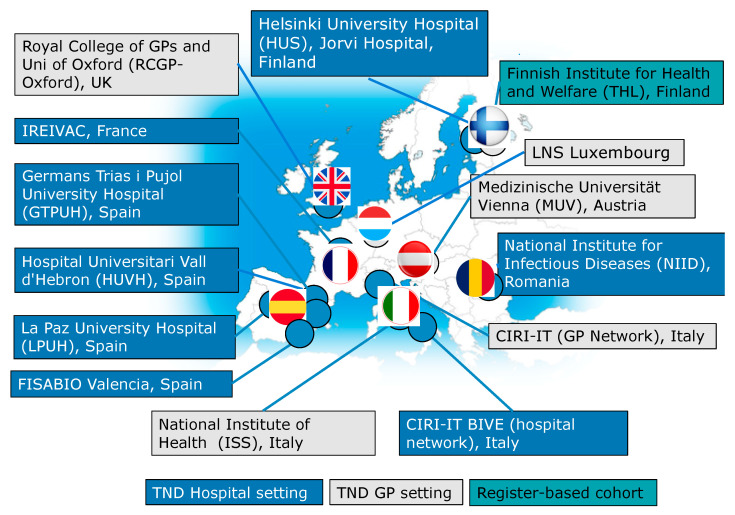
DRIVE study sites 2019/2020 map. Fourteen study sites from a total of eight different European countries collected data for DRIVE during the 2019/2020 influenza season. In total, there were 19 hospitals and 388 GPs for the TND studies and more than 1.5 million subjects for the register-based cohort study.

In 2019/2020, DRIVE continued its expansion and included 14 sites from eight different European countries. For the 2019/2020 season, four primary-care-based test-negative design (TND) studies (Austria, England, Italy and the UK), eight hospital-based TND studies (Finland, France, Italy, Romania, Spain) and one register-based cohort study (Finland) were conducted (Table 1, Figure 1). The COVID-19 outbreak impacted influenza surveillance; thus, the study period for the main analysis was truncated. The pandemic and the subsequent lockdown measures interfered with and capped an already mild influenza circulation and impacted data collection within DRIVE study sites. Despite all these challenges, precise brand-specific estimates were obtained from the pooled TND studies in DRIVE, in addition to those from the population-based cohort study [2]. 

## 2. New Challenges Encountered at DRIVE Study Platform Level Due to COVID-19 Pandemic

COVID-19 Europe-wide circulation started in February 2020 and overlapped with the 2019/2020 influenza season only for a few weeks between the end of February and beginning of March [6]. Thus, there was not an actual co-epidemic of influenza and SARS-CoV-2 in the 2019/2020 season in Europe; however, COVID-19 and influenza are both respiratory infectious diseases with similar clinical presentation. As a consequence, the COVID-19 pandemic directly impacted influenza surveillance systems and data collection at the DRIVE study sites (this will be described in detail in the next sections). 

First, the COVID-19 health crisis has pushed healthcare systems to the limit of their capacities or beyond and has changed health-care–seeking behaviors for people presenting influenza like illness (ILI) symptoms.

Second, measures taken by public health authorities and governments to reduce virus spread, such as containment, social distancing and lockdown, for COVID-19 dramatically affected the transmission of other infectious diseases such as influenza. 

Finally, the COVID-19 pandemic forced several sites to stop the inclusion of influenza cases in early March, mostly due to lockdown measures, safety measures within the hospitals, COVID-19–related work overload and clinical staff being diverted to COVID-19– related tasks (e.g., SARS-CoV-2 testing and clinical management of COVID-19 patients). Overall, data collection for DRIVE and testing for influenza was de-prioritized, and as a consequence, the last influenza swabs of the season were collected in mid-March of 2020. The DRIVE study period for the 2019/2020 season effectively ended in week 12 of 2020 in all study sites (as the study period end was defined as the week prior to the first of two consecutive weeks when no influenza viruses were detected) [2]. 

Altogether, the previous points highlighted DRIVE’s necessity to account for the COVID-19 pandemic in the IVE study platform.

### 2.1. DRIVE IVE Study Platform and COVID-19: Actions Taken in the 2019/2020 Season

DRIVE updated both the Statistical Analysis Plan (SAP) and the IVE results report for the 2019/2020 season to account for the altered COVID-19 case management and influenza surveillance among study sites (i.e., study period and sensitivity analysis).

**Figure 2:**
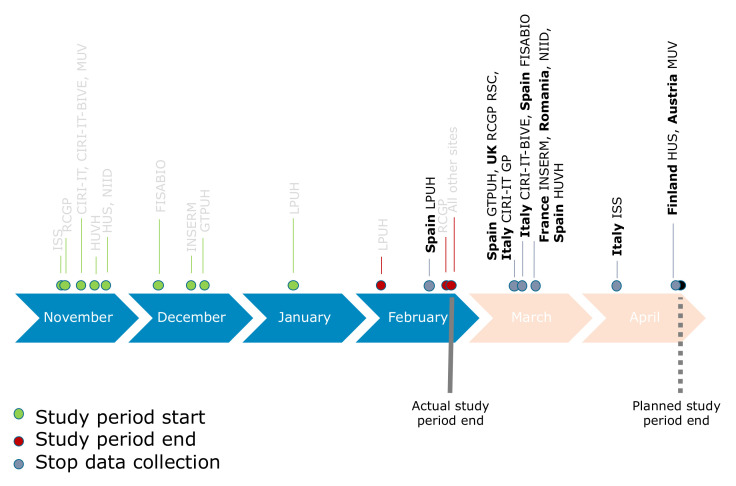
New study period adjusted due to COVID-19 pandemic. Study period for main analysis was considered until 29 February 2020; sensitivity analysis considered data collected until 30 April 2020. However, the actual study period end (defined as first week of two subsequent weeks of no influenza cases detected) was in week 12 of 2020 at all DRIVE sites, indicating there was no more influenza circulating even at the sites that did still collect data beyond end of February. Only showing TND studies.

At the site level, the end of the study period was defined as either the week prior to the first of two consecutive weeks when no influenza viruses were detected, or the previously set end of the study period (30 April 2020), whichever occurred first. As illustrated in Figure 2 and described in Table 2, several sites stopped collecting data by the beginning of March. Moreover, the influenza season was shorter than expected due to the lockdown, social distancing and other COVID-19 protective measures; thus, no influenza-positive swabs were collected beyond week 12 of 2020. Therefore, the end of the study period for the main analysis was established as 29 February 2020 (two months earlier than the originally established date of 30 April 2020).

A sensitivity analysis that included data up to 30 April was performed. All local study periods effectively ended before 30 April (Figure 2), but no more influenza positive tests were reported after week 12. Precise IVE estimates in the main analysis were similar to the estimations obtained from the sensitivity analysis with the extended study period, and two additional estimates with a confidence interval (CI) width of <40% were obtained [2,7].

DRIVE study sites did not exclude COVID-19 cases from the DRIVE study for the 2019/2020 season. This is in line with previous DRIVE seasons, in which test results for respiratory viruses other than influenza did not impact the inclusion of subjects. Thus, COVID-19 patients were only excluded from DRIVE study if they met the regular DRIVE exclusion criteria. However, data on the COVID-19 status of subjects recruited was not collected as this was not foreseen in the DRIVE protocol for the 2019/2020 season. 

Several DRIVE study sites implemented a different triage protocol in response to SARS-CoV-2 emergence, and all SARI cases arriving to the hospital were tested first for SARS-CoV-2; if results were negative, later tests were performed for other respiratory viruses such as influenza (Table 2). This new triage strategy was not expected to significantly reduce the number of influenza cases captured in the DRIVE dataset in 2019/2020, as very few cases of co-infection of influenza/SARS-CoV-2 were reported at the DRIVE sites that did test swabs for both viruses; this was due the minimal overlap between the influenza season and the emergence of the SARS-CoV-2 pandemic. 

### 2.2. DRIVE Study Sites Experience: Management of the DRIVE Study during the COVID-19 Pandemic

This section provides more details on the experiences of two DRIVE study sites in 2019/2020 in terms of management of the DRIVE study during the COVID-19 pandemic as well as the challenges faced and solutions designed to overcome these challenges. Each of the study sites below approached the DRIVE study during the COVID-19 outbreak in different and unique ways: CIRI-IT, in Italy, had to deal with an intense COVID-19 outbreak, whereas HUS Jorvi Hospital in Finland faced a moderate COVID-19 outbreak.

#### 2.2.1. Interuniversity Research Center on Influenza and Other Transmissible Infections (CIRI-IT, Italy)

CIRI-IT (Interuniversity Research Center on Influenza and other Transmissible Infections) participates in DRIVE: (1) through a hospital network composed of five Italian hospitals and (2) through a network of 35 physicians (25 GPs and 10 pediatricians) in two Italian regions, Liguria and Veneto. The first local case of COVID-19 in Italy was confirmed on 22 February, and over the following days, cases were reported in several other regions. This emergency led to a national lockdown on 9 March 2020.

Until the first week of March 2020, the data collection for DRIVE was not affected by the COVID-19 emergency. However, later in March, the COVID-19 emergency affected the enrollment of SARI cases in all CIRI-IT hospitals and the enrollment of ILI cases by the CIRI-IT physicians and it resulted in an end to data collection for DRIVE (last SARI swab collected for DRIVE was 15 March 2020 in Siena hospital and last ILI swab was collected on 13 March 2020).

During the initial stages of the COVID-19 pandemic, in order to minimize the possibility of contagion, ILI cases enrolled by the physicians’ network and the patients admitted with SARI in the hospitals were regarded as “suspected COVID-19 cases”. These subjects followed a new clinical pathway, which made it difficult to (a) collect useful information for filling in the questionnaire required by the DRIVE project, and (b) carry out PCR tests for the presence of influenza. These patients were initially subjected to laboratory tests for SARS-CoV-2 only. However, after testing for SARS-CoV-2, the swab sample from each patient was frozen, and testing for the detection of influenza was performed on the frozen samples later on. Accordingly, the collection date of the last swab positive for influenza was actually 13 March 2020 for the hospital study and 4 March 2020 for the primary care study, but the testing for influenza of those frozen samples was performed in April. In addition, patients and physicians were also contacted at a later stage to obtain the information necessary for the DRIVE study.

#### 2.2.2. Helsinki University Hospital, Jorvi Hospital (HUS, Finland)

In Finland, the COVID-19 pandemic remained moderate in comparison with countries such as Italy and Spain. DRIVE research collaborators in HUS were able to keep their staff working for DRIVE and did not stop collecting data for the DRIVE study, finishing data collection on 30 April, as initially scheduled. However, after the emergence of COVID-19 and the subsequent lockdown measures, influenza cases nearly disappeared (and, previously, the influenza season in Finland was also considered very mild). As a result, the number of influenza patients fell short of what was expected (33 vs. 74 last season). The influenza cases recruited for the DRIVE study in the 2019/2020 season were also included in a prospective observational study comparing the clinical characteristics and outcomes of hospitalized adult COVID-19 and influenza patients [8].

Suspected COVID-19 patients were recruited in the DRIVE study, as they fulfilled the SARI criteria, and the study site protocol accounted for SARS-CoV-2 testing. However, as SARS-CoV-2 testing was prioritized over influenza, sample logistics were disturbed and, in many cases, only SARS-CoV-2 PCR testing was performed. Most of the samples tested for SARS-CoV-2 were stored for later re-analysis for influenza. Unfortunately, a small percentage of the stored samples were lost and consequently not re-analyzed for influenza.

The HUS study site faced several other challenges due to the COVID-19 pandemic. For instance, patients could not be visited due to strict isolation, and thus it was difficult to obtain their informed consent. To overcome this problem, HUS implemented the strategy of oral witnessed consent. Oral consent was taken on the phone (on speaker, so the witness heard the conversation) or from outside the isolation area. The witness had to be a member of the hospital staff who was not included in the study team. The study nurse and the witness signed the consent form. Oral informed consent given by the next of kin was used in the same manner.

Oral witnessed consent was a form of consent inbuilt to the HUS study protocol and was approved by their ethics committee. This form of consent was initially included as there was the possibility of influenza patients in the ICU who might not be able to give their written informed consent due to being too ill to write, but who could still give their oral consent. As it turned out, it was mostly needed for including seriously ill or isolated COVID-19 patients. During the COVID-19 epidemic, witnessed oral consent was very practical both for avoiding the risk of transmission and for saving PPE sorely needed by hospital staff.

## 3. Anticipating the Impact of COVID-19 on the IVE Studies for the Upcoming Influenza Seasons: Next Steps

### 3.1. DRIVE Study Platform Adaptation for COVID-19 for 2020/2021 Season

In the current COVID-19 pandemic context, it is expected that influenza and SARS-CoV-2 viruses will co-circulate partially or entirely during the 2020/2021 season [9], so the impact on the DRIVE study will likely be higher than in the 2019/2020 season. The change in health-seeking behavior and further lockdown policies due to subsequent COVID-19 pandemic waves in Europe, differences in vaccine recommendations and distribution, the standard of care and access to flu vaccination [10], and further elements yet to be explored, will undoubtedly generate bias in IVE estimates and/or affect the operational execution of the IVE study platform. 

After consultation with EMA and IMI in April 2020, the DRIVE consortium decided to account for COVID-19 in its study documents (protocols, SAP, etc.) and operational procedures for the 2020/2021 season. Since then, DRIVE is closely tracking the evolution of the pandemic and liaising with the study sites to rapidly adapt to this ever-changing situation.

Consequently, DRIVE will use the 2020/2021 season as a feasibility study for discussion on COVID-19 components that can be implemented in the study platform in order to better understand how COVID-19 would impact the brand-specific IVE. A few points should be considered:

- SARS-CoV-2 virus infection could potentially be a confounder for the association between influenza vaccination and influenza disease [11].
- In potential co-circulation of influenza and SARS-CoV-2, efforts to increase rates of influenza vaccination are important to reduce the additional influenza-related burden, particularly among groups at high risk of influenza complications and severe COVID-19 disease. Thus, COVID-19 might influence the influenza vaccine recommendations or its acceptance, increasing the differences between the vaccinated and unvaccinated [12].
- There may also be differences in healthcare-seeking behavior for influenza vs. COVID-19 (e.g., at-risk individuals and severity of the new disease) leading to under- or over-estimation of IVE.
- Co-infection may affect clinical outcomes and individual response to vaccine-derived protection for influenza.
- The standard of care and access to influenza vaccination might be impacted in the case of SARS-CoV-2 virus circulation.

### 3.2. Adaptation of DRIVE TND Protocol to Include COVID-19 Components

DRIVE has updated its TND protocol for the 2020/2021 season in order to account for several of the COVID-19 particularities mentioned above. First, two COVID-19 related objectives were added to the protocol. A secondary objective is to estimate the COVID-19 impact on IVE within the adults/older adults in a hospital setting, given the COVID-19 epidemiology. As exploratory objective is to describe clinical signs and symptoms as well as laboratory features, around the point of admission, among hospitalized COVID-19 cases as compared to influenza cases.

Moreover, the following variables will be collected across the study sites:

- COVID-19 positivity in the current season (RT-PCR test)
- Date of COVID-19 test
- COVID-19 positivity in the previous season
- Use of COVID-19 treatments and type (e.g., antivirals)
- Clinical symptoms to distinguish between influenza and COVID-19, such as anosmia, ageusia, etc.
- Co-morbidities to identify risk-specific groups for COVID-19
- Morbidity related to COVID-19

The collection of these variables is not mandatory for DRIVE study sites, but preferrable. Table 3 indicates which study sites will be able to collect COVID-19–related variables in the 2020/2021 season.

### 3.3. DRIVE Study Site Operations and Data Collection for the 2020/2021 Season

**Figure 3:**
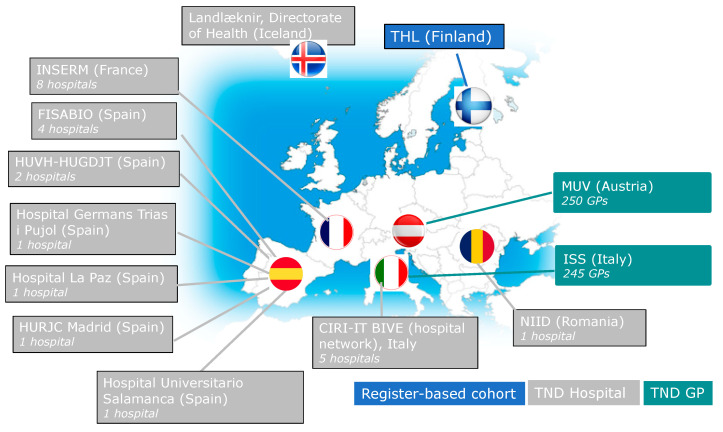
DRIVE study sites for the 2020/2021 season: 13 study sites from 7 different European countries.

Triage strategies will differ among sites in the 2020/2021 season (Figure 3, Table 3). The majority of the DRIVE study sites have established a common triage for all SARI/ILI cases, which will be tested simultaneously for both influenza and SARS-CoV-2 in multiplex RT-PCR. However, other sites will prioritize SARS-CoV-2 testing, and only those swabs testing negative for SARS-CoV-2 will be stored for subsequent influenza testing.

## 4. DRIVE beyond Influenza: COVID-19 Vaccine Effectiveness Studies

DRIVE has envisioned since its very beginning a vaccine effectiveness (VE) platform beyond influenza, which will eventually be able to evaluate the effectiveness of vaccines for other respiratory diseases. The urgency of the COVID-19 pandemic and the imminence of the new COVID-19 vaccines has pushed DRIVE to move quickly and to leverage DRIVE assets to set up a COVID-19 VE platform as soon as possible. 

Because of the uncertainty of the allocation modalities, distribution and timing of early COVID-19 vaccine candidates in Europe, it will be very challenging to predict the level of uptake and ensure sufficient a sample size for VE studies [13,14]. This will necessitate the set-up of large study network with wide geographical coverage. In addition, COVID-19 and influenza are both respiratory infectious diseases with similar clinical symptoms, respiratory specimens and laboratory tests as well as potentially having time periods leading to an overlap of surveillance [15,16]. Biases may also be expected if they are not jointly considered. 

**Figure 4:**
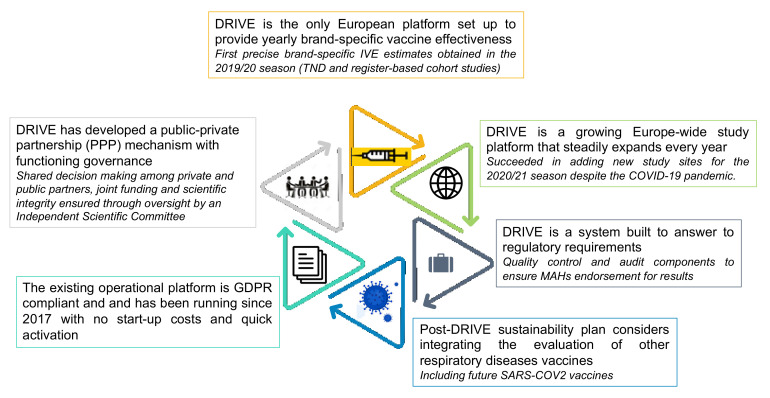
DRIVE key assets and achievements. Since its inception in 2017, DRIVE has achieved several milestones and developed key assets that make it a unique study platform for brand-specific VE studies in Europe. DRIVE has developed methods to pool brand-specific IVE data.

As presented in Figure 4, DRIVE has developed several assets that can be leveraged to readily encompass SARS-CoV-2 in addition to influenza. DRIVE’s existing brand-specific study platform, governance, tools and methods could be readily adapted to add COVID-19–specific components, allowing for limited start-up cost and increased efficiency. Thus, within the current vaccines’ post-marketing landscape, DRIVE could potentially provide the base of a new study platform. leading the COVID-19 vaccine effectiveness monitoring field.

## 5. Conclusions

DRIVE was able to obtain precise brand-specific IVE estimates in the 2019/2020 season despite the start of the COVID-19 pandemic during the influenza season [2]. This achievement reflects the soundness of the DRIVE study network and how well DRIVE study sites managed the challenges posed by the COVID-19 pandemic, which have been reflected in the present paper. The pandemic and the subsequent lockdown measures interfered with and capped an already mild influenza circulation and impacted data collection within DRIVE study sites. 

COVID-19 will also have a significant impact on the DRIVE study platform for the 2020/2021 season, both in terms of epidemiology and data collection at DRIVE study sites. In order to better interpret IVE, the DRIVE generic protocol for TND studies and SAP has been adapted to encompass several COVID-19 components regarding the operational aspects of data collection and analysis. These adaptations aim to estimate the COVID-19 impact on IVE and to compare clinical and laboratory features of COVID-19 and influenza cases at the time of hospital admission. The COVID-19 and influenza testing strategy at the study sites has yet to be fully understood, as healthcare-seeking behaviors, triage strategies and testing pathways have been adapted in many European countries. A good understanding of all the COVID-19 adaptations that DRIVE sites have implemented will be important to accurately describe the study population. Consequently, DRIVE is currently studying the feasibility of implementing COVID-19 surveillance elements in the DRIVE study platform. 

In the future, DRIVE proposes to leverage the COVID-19 experience during the 2019/2020 and 2020/2021 seasons to design its proposal for a post-DRIVE study platform for influenza and other potential respiratory diseases (e.g., COVID-19, respiratory syncytial virus (RSV)) by developing a proof of concept. This proof of concept will consider the feasibility of adapting the DRIVE influenza study platform for the evaluation of future COVID-19 vaccines and will propose a PPP model to create a common study platform to monitor the vaccine effectiveness of several respiratory infectious diseases.

## Figures and Tables

**Table 1:** Evolution of DRIVE study platform in the years since its start in 2017. Specified information includes the number of participant study sites/European countries; total number of general practitioners (GP)/hospitals participating in the test-negative design (TND) studies; number of influenza-like illness (ILI) cases, severe acute respiratory infection (SARI) cases and laboratory-confirmed influenza (LCI) recruited in the TND; figures for the register-based cohort studies and influenza vaccine brands captured in DRIVE dataset.

	Season 2017/2018	Season 2018/2019	Season 2019/2020
Comments	Pilot season, severe influenza season	Mild influenza season	COVID-19 impact (shorter study period) and mild influenza season
Study platform	5 sites 4 countries	13 sites 7 countries	14 sites 8 countries
Countries	Austria, Finland, Italy and Spain	Added: Luxembourg, Romania and United Kingdom	Added: France
TND Hospital	4 hospitals 2.882 SARI 436 LCI	12 hospitals 4.884 SARI 1.442 LCI	19 hospitals 4.121 SARI 1.296 LCI
TND GP	361 GP 4.343 ILI 2.419 LCI	378 GP 5.008 ILI 2.159 LCI	388 GP 4.958 ILI 2.235 LCI
Register-based cohort	1.326.097 subjects 5.708 LCI	1.516.946 subjects 6379 LCI	1.550.000 subjects 2427 LCI
Influenza vaccine brands	11 on market 10 in DRIVE dataset	10 on market 7 in DRIVE dataset	11 licensed 8 in DRIVE dataset

**Table 2:** DRIVE study sites: COVID-19 impact on data collection, triage and testing strategies during the 2019/20 season. * Children: 6 months to 17 years old recruited in 2019/2020 season; ** Adults: 17 to 65 years old recruited in 2019/2020 season; *** Older adults: >65 years old recruited in 2019/2020 season.

DRIVE Study Sites 2019/2020	Characteristics	Was Testing for Influenza Impacted?	Did Triage Strategy Change?	Date of Last Swab Positive for Influenza	DRIVE Data Collection
Fisabio (Spain)	Hospital setting (4 hospitals) 19 children * 157 adults ** 486 older adults ***	No, only samples that tested negative for influenza (and the other respiratory viruses included in VAHNSI multiplex PCR) were frozen and re-analyzed for SARS-CoV-2.	No	13 March 2020	Stopped (13 March 2020) The collection of samples for DRIVE stopped in early March to prevent risk of infection of nurses, as well as because of the added complexity to access isolated patients for informed consent/questionnaires.
Hospital Germans Trias i Pujol (Spain)	Hospital setting (1 hospital) 25 children * 68 adults ** 89 older adults ***	No, all samples from SARI patients were tested for both SARS-CoV-2 and influenza virus with multiplex PCR.	No	12 March 2020	Continued data collection until 30 April 2020 (DRIVE initial data cut-off) Since there was one staff member dedicated exclusively to reviewing and entering the DRIVE data, the study site could collect all the information needed for the project.
Hospital La Paz (Spain)	Hospital setting (1 hospital) 15 adults ** 21 older adults ***	Yes, samples from all SARI cases admitted to the hospital were only tested for SARS-CoV-2 and no subsequent PCR testing for influenza was performed.	Yes, all SARI cases arriving to the hospital were managed as COVID-19 cases, so they were isolated and immediately tested for SARS-CoV-2 (and not for influenza).	24 February 2020	Stopped (first week of March)
Hospital Universitari Vall d’Hebron (Spain)	Hospital setting (1 hospital) 78 children * 110 adults ** 100 older adults ***	No, every patient suspected of COVID-19 was tested for both SARS-CoV-2 and influenza virus.	No, screening of SARI cases was not affected during the COVID-19 pandemic.	16 March 2020	Continued data collection until 30 April 2020 (DRIVE initial data cut-off)
I-REIVAC (France)	Hospital setting (5 hospitals) 134 adults ** 246 older adults ***	No, however, the FLUVAC study protocol did not implement routinely SARS-Cov-2 testing, so patients hospitalized with SARI were not tested for SARS-CoV-2 at admission and the information on SARS-CoV-2 positivity was initially missing. However, all samples were stored to be re-analyzed for SARS-CoV-2 later on.	Yes, the algorithm for hospital screening of admitted patients was different between eligible SARI and suspected COVID-19 individuals, who were isolated. Thus, clinical research assistants of the IREIVAC centers did not get access to the samples of suspected COVID-19 patients for influenza testing.	16 March 2020	Stopped (20 March 2020) The French national public health agency stopped influenza surveillance at the country level by mid-March and switched to specific COVID-19 monitoring.
ISS (Italy)	Primary Care setting (245 GP) 938 children * 863 adults ** 119 older adults ***	Yes, simultaneous test for influenza and SARS-CoV-2 was not implemented, so ILIs were tested only for SARS-CoV-2 and not for influenza. Samples were stored and re-analyzed later on for influenza.	Yes, ILI cases arriving to the GP during the COVID-19 pandemic were considered COVID-19 suspects and tested only for SARS-CoV-2.	7 April 2020	Continued data collection until 30 April 2020 (DRIVE initial data cut-off)
CIRI-IT BIVE (Italy)	Hospital setting (5 hospitals) 770 children * 296 adults ** 584 older adults ***	Yes. At the beginning of the COVID-19 pandemic (March 2020), the impossibility of performing the simultaneous test for influenza and SARS-CoV-2 was real. So, all SARI cases were initially tested for SARS-CoV-2, samples stored, and then re-analyzed for influenza.	Yes, ILI cases arriving to the emergency department during the COVID-19 pandemic were considered COVID-19 suspects and tested initially only for SARS-CoV2.	13 March 2020	Stopped (15 March 2020)
CIRI-IT GP (Italy)	Primary Care setting (35 GP) 698 children * 524 adults ** 146 older adults ***	Yes. At the beginning of the COVID-19 pandemic (March 2020), the impossibility of performing the simultaneous test for influenza and SARS-CoV-2 was real. So, all ILI cases were initially tested for SARS-CoV-2, samples stored, and then re-analyzed for influenza.	Yes, ILI cases consulting the physicians during the COVID-19 pandemic were considered COVID-19 suspects, and to minimize the possibility of contagion, physicians had limited access to their clinics to patients with ILI. ILI cases were initially tested for SARS-CoV-2, samples stored, and then re-analyzed for influenza.	4 March 2020	Stopped (13 March 2020)
MUV (Austria)	Primary Care setting (96 GP) 639 children * 673 adults ** 47 older adults ***	No, Austria efficiently switched from influenza to SARS-CoV-2 surveillance and MUV implemented the SARS-CoV-2 testing into the sentinel system on 24 February 2020, meaning that each sample was tested both for influenza and SARS-CoV-2 by PCR.	Yes, with the beginning of the COVID-19 pandemic in Austria, the ICU units were separated in COVID ICUs and non- COVID ICUs, preventing nosocomial infections.	13 March 2020	Continued data collection until 30 April 2020 (DRIVE initial data cut-off) Routine influenza screening at the primary care level almost stopped, as each patient requiring ICU admission got tested for SARS-CoV-2 only. This testing was performed in the respective hospitals and not at the GPs’; therefore, MUV was not able to get these samples for influenza testing.
NIID (Romania)	Hospital setting (1 hospital) 500 children * 221 adults ** 78 older adults ***	In the beginning of the COVID-19 pandemic all samples from SARI patients were tested for both SARS-CoV-2 and influenza simultaneously. However, from 22 March, SARI cases were only tested for influenza after a positive test for SARS-CoV-2. From 22 March to 30 April 2020, NIID only identified 1 coinfection of SARS-CoV-2 + influenza B.	In the initial stages of the COVID-19 pandemic, NIID was the designated facility for SARS-CoV-2 diagnosis and surveillance in Romania. All SARI cases admitted to the hospital were tested for both SARS-CoV-2 and influenza, so the recruitment of cases for DRIVE studies did not stop.	16 March 2020	Continued data collection until 30 April 2020 (DRIVE initial data cut-off) As of 22 March 2020, NIID switched to a COVID-19 hospital, so only COVID-19 cases were admitted. The screening for influenza was only performed for patients positive for SARS-CoV-2. Thus, the vast majority of the influenza cases were admitted to other hospitals.

**Table 3:** DRIVE study sites: triage and testing strategies as well as ability to collect information on COVID-19 variables for the 2020/21 season.

DRIVE Study Sites 2020/2021	Characteristics	Planned Triage Influenza/COVID-19	Laboratory Testing	Possibility to Collect COVID-19 Variables
Fisabio (Spain)	Hospital setting (4 hospitals)	Common triage (all ILI/SARI cases admitted).	Simultaneous RT-PCR testing for SARS-CoV-2 and influenza, from the same swab.	Yes COVID-19 positivity in the current season (RT-PCR test) Time of COVID-19 test COVID-19 positivity in the previous season Use of COVID-19 treatments: dexamethasone, remdesivir, favpiravir, lopinavir/ritonavir and non-steroidal anti-inflammatory drugs Clinical symptoms to distinguish between influenza and COVID-19 Co-morbidities to identify risk-specific groups for COVID-19 Morbidity related to COVID-19 (pneumonia, etc.)
Hospital Germans Trias i Pujol (Spain)	Hospital setting (1 hospital)	Common triage (all SARI cases admitted).	Simultaneous RT-PCR testing for SARS-CoV-2 and influenza, not yet established if it will be from the same swab.	Yes COVID-19 positivity in the current season (RT-PCR test) Time of COVID-19 test COVID-19 positivity in the previous season Use of COVID-19 treatments: according to hospital’s protocols Clinical symptoms to distinguish between influenza and COVID-19 Co-morbidities to identify risk-specific groups for COVID-19 Morbidity related to COVID-19 (pneumonia, etc.)
Hospital Universitari Vall d’Hebron (Spain)	Hospital setting (2 hospitals)	Common triage (all SARI cases admitted). This study site avoids many of the problems generated by prospective studies and risk of COVID-19 infections by selecting the cases and controls from the influenza surveillance electronic system of Catalonia, so there will be no problem accessing patient data if isolated.	Simultaneous RT-PCR testing for SARS-CoV-2 and influenza, from the same swab.	Yes COVID-19 positivity in the current season (RT-PCR test) Time of COVID-19 test COVID-19 positivity in the previous season
CIRI-IT (Italy)	Hospital setting (5 hospitals)	Common triage of all SARI cases admitted.	Simultaneous RT-PCR testing for SARS-CoV-2 and influenza, from the same swab.	Yes COVID-19 positivity in the current season (RT-PCR test) Time of COVID-19 test Clinical symptoms to distinguish between influenza and COVID-19 Co-morbidities to identify risk-specific groups for COVID-19 Morbidity related to COVID-19 (pneumonia, etc.)
MUV (Austria)	Primary Care setting (250 GP)	Different triage for ILI cases suspected case of COVID-19: if a suspected COVID-19 case, patient stays at home and physicians come to take the swab. If ILI case is suspected of influenza, normal GP appointment. In all ILI cases, swabs are tested for both influenza and SARS-CoV-2.	Simultaneous RT-PCR testing for SARS-CoV-2 and influenza, from the same swab.	Yes, but limited to Clinical symptoms to distinguish between influenza and COVID-19 Co-morbidities to identify risk-specific groups for COVID-19
IREIVAC (France)	Hospital setting (8 hospitals)	Common triage of all SARI cases admitted.	Simultaneous RT-PCR testing for SARS-CoV-2 and influenza, from the same swab.	Yes COVID-19 positivity in the current season (RT-PCR test) Time of COVID-19 test COVID-19 positivity in the previous season Use of COVID-19 treatments: antivirals, monoclonal antibodies/IL-6 blockers, chloroquine, hydroxychloroquine, antibiotics, corticoids Clinical symptoms to distinguish between influenza and COVID-19: anosmia, ageusia, etc. Co-morbidities to identify risk-specific groups for COVID-19 Morbidity related to COVID-19 (pneumonia, etc.)
Directorate of Health (Iceland)	Hospital and primary care settings	Common triage at the primary care and hospital level.	Simultaneous RT-PCR testing for SARS-CoV-2 and influenza, from the same swab.	Yes COVID-19 positivity in the current season (RT-PCR test) Time of COVID-19 test
NIID (Romania)	Hospital setting (1 COVID-19– only hospital)	Not common triage of SARIs arriving to the hospital: only SARS-CoV-2 positives are admitted to the hospital (as it is a COVID-19–only hospital). SARS-CoV-2 negatives are diverted to other hospitals.	Simultaneous RT-PCR testing for SARS-CoV-2 and influenza. Swabbing case-by-case: if the patient is already confirmed as having COVID-19, another swab will be taken for influenza.	Yes COVID-19 positivity in the current season (RT-PCR test) Time of COVID-19 test COVID-19 positivity in the previous season Use of COVID-19 treatments: all administered Clinical symptoms to distinguish between influenza and COVID-19 Co-morbidities to identify risk-specific groups for COVID-19 Morbidity related to COVID-19 (pneumonia, etc.)
Hospital U. Salamanca (Spain)	Hospital setting (1 hospital)	Common triage of all SARI cases admitted.	All subjects are tested for both SARS-CoV-2 and influenza, but testing for influenza will be done at the end of the season, from the same swab.	Yes COVID-19 positivity in the current season (RT-PCR test) Time of COVID-19 test COVID-19 positivity in the previous season Use of COVID-19 treatments Clinical symptoms to distinguish between influenza and COVID-19 Co-morbidities to identify risk-specific groups for COVID-19 Morbidity related to COVID-19 (pneumonia, etc.)
Hospital U. La Paz (Spain)	Hospital setting (1 hospital)	Common triage of all SARI cases admitted.	Swab will initially be tested for SARS-CoV2, and subsequently tested for influenza and other respiratory viruses (same swab).	Yes COVID-19 positivity in the current season (RT-PCR test) Time of COVID-19 test COVID-19 positivity in the previous season Use of COVID-19 treatments: dexamethasone, remdesivir Clinical symptoms to distinguish between influenza and COVID-19 Co-morbidities to identify risk-specific groups for COVID-19 Morbidity related to COVID-19 (pneumonia, etc.)

## Data Availability

The results from the DRIVE IVE studies (seasons 2017/2018, 2018/2019 and 2019/2020) are available in the DRIVE website via the following link: https://www.drive-eu.org/index.php/results/.

## Abbreviations

CIRI-IT	Interuniversity Research Center on Influenza and other Transmissible Infections
COVID-19	Coronavirus disease 2019
DRIVE	Development of Robust and Innovative Vaccine Effectiveness
ECDC	European Centre for Disease Prevention and Control
EMA	European Medicines Agency
EU	European Union
FISABIO	Fundación para el Fomento de la Investigación Sanitaria y Biomédica de la Comunitat Valenciana
GP	General practice
GTPUH	Germans Trias I Pujol University Hospital
HUS	Helsinki University Hospital, Jorvi Hospital
HUVH	Hospital Universitari Vall D’Hebron
ICU	Intensive care unit
ILI	Influenza-like illness
IMI	Innovative Medicines Initiative
IREIVAC	Innovative Clinical Research Network in Vaccinology
IVE	Influenza vaccine effectiveness
LCI	Lab-confirmed influenza
LPUH	La Paz University Hospital
MUV	Medical University Vienna
NIID	National Institute for Infectious Disease “Prof. Dr. Matei Bals”
PPE	Personal protective equipment
RSV	Respiratory syncytial virus
RT-PCR	Reverse-transcriptase polymerase chain reaction
SAP	Statistical analysis plan
SARI	Severe acute respiratory illness
SARS-CoV-2	Severe acute respiratory syndrome coronavirus 2
TND	Test-negative design
VAHNSI	Valencia Hospital Surveillance Network for the Study of Influenza and Respiratory Viruses Disease
